# An AST-ELM Method for Eliminating the Influence of Charging Phenomenon on ECT

**DOI:** 10.3390/s17122863

**Published:** 2017-12-09

**Authors:** Xiaoxin Wang, Hongli Hu, Huiqin Jia, Kaihao Tang

**Affiliations:** 1 Key Laboratory of Education Ministry for Photoelectric Logging and Detecting of Oil and Gas, Xi’an Shiyou University, Xi’an 710065, China; jiahq@xsyu.edu.cn; 2 State Key Laboratory of Electrical Insulation and Power Equipment, Xi’an Jiaotong University, Xi’an 710049, China; hlhu@mail.xjtu.edu.cn (H.H.); mrerr07@stu.xjtu.edu.cn (K.T.)

**Keywords:** electrical capacitance tomography (ECT), charging phenomenon, extreme learning machine, adaptive soft-thresholding

## Abstract

Electrical capacitance tomography (ECT) is a promising imaging technology of permittivity distributions in multiphase flow. To reduce the effect of charging phenomenon on ECT measurement, an improved extreme learning machine method combined with adaptive soft-thresholding (AST-ELM) is presented and studied for image reconstruction. This method can provide a nonlinear mapping model between the capacitance values and medium distributions by using machine learning but not an electromagnetic-sensitive mechanism. Both simulation and experimental tests are carried out to validate the performance of the presented method, and reconstructed images are evaluated by relative error and correlation coefficient. The results have illustrated that the image reconstruction accuracy by the proposed AST-ELM method has greatly improved than that by the conventional methods under the condition with charging object.

## 1. Introduction

Electrical capacitance tomography (ECT) is one of the most developed tomographic modalities among many industrial process tomography techniques due to its advantages of noninvasion, low-cost, nonradiation and good safety performance [1,2,3,4,5]. The applications of ECT in gas–liquid, gas–solid and liquid–solid two-phase flows measurement are gaining wide acceptance in laboratory research and industrial applications [6,7,8]. In the pneumatically conveying process of gas–solid flow, particle electrification occurs due to friction, collision and separation between particles and between particles and the pipe wall [9,10,11], and the charged particle induces a certain amount of charge on the detection electrode of ECT and affect the measurement accuracy of ECT. 

Some scholars have studied the influence of charged particle on capacitance measurement. Matsusaka et al. [11,12] summarized the basic concepts of contact charging and formulated the variation of the particle charging caused by repeated impacts on a wall. The theory is extended to the particle charging in gas–solids pipe flow, where each particle has a different amount of charge; the distribution of the particle charge is also analyzed theoretically. Kanazawa et al. [13] researched the generation and accumulation mechanism of the charge on the inner surface of a powder transport pipe and drew a conclusion that the charge density and polarity actually depended on the successive number of tests and the pipe material. Gao et al. [14] investigated the electrostatic effect on alternating current (AC)-based ECT by simulations and experiments, and an ECT combined with an electrostatic tomography system (EST) was used to verify theoretical analysis. These work proved the fact that particle charge has influences on the capacitance measurement. However, little research has addressed the method to eliminate the influence of charged particle on capacitance measurement. Jian Li et al. [15] analyzed the influence of particle electrification on AC-based capacitance measurement circuit with helical electrodes and proposed a modified AC-based capacitance measurement circuit to eliminate the influences. Considering the application of ECT in gas–solid two-phase flow measurement is more and more widely, more studied are still essential to deal with this phenomenon.

Many studies have researched the inverse problem including linear back projection (LBP), Landweber iteration, algebraic reconstruction technique (ART), Tikhonov regularization methods, simultaneous iterative reconstruction technique (SIRT), regularized Gauss–Newton algorithm, support vector machine (SVM) and back propagation (BP) neural network [16,17,18,19,20,21,22]. The algorithms mentioned above can be classified into three types: one-step algorithm, iteration algorithm and neural network algorithm. Compared with other algorithms, the neural network algorithm can construct a mapping relationship between the input capacitance value and the image gray-scale value by using machine learning but not an electromagnetic-sensitive mechanism. The performance of the neural network algorithm is investigated for the image reconstruction of charging object in this paper. Due to the traditional neural network image reconstruction algorithm has the main defect of slow calculation, the extreme learning machine (ELM) with the fast learning speed is applied to the image reconstruction. The extreme learning machine (ELM) [23,24] proposed by Guang-Bin Huang in 2004 is a new neural network algorithm for single-hidden layer feed-forward neural networks (SLFNs). ELM has been widely used in pattern recognition, fault diagnosis and classification. ELM provides high generalization ability at an extremely fast learning speed. However, due to the sparsity and ill-posedness of ECT, ELM should be further researched to improve the robustness of image reconstruction.

In order to provide a stable nonlinear mapping model between the capacitance values and medium spatial distribution with good robustness, this paper presents an improvement algorithm AST-ELM which combined extreme learning machine (ELM) with adaptive soft-thresholding (AST) method. Experiments are carried out on a pulley rig with triboelectrification to validate the performance of the presented method. Both simulated and measured results are compared with conventional methods LBP and Landweber algorithms in two aspects: relative error and correlation coefficient.

## 2. Effects of Electrification Phenomenon on ECT Using Simulation Analysis

**Figure 1:**
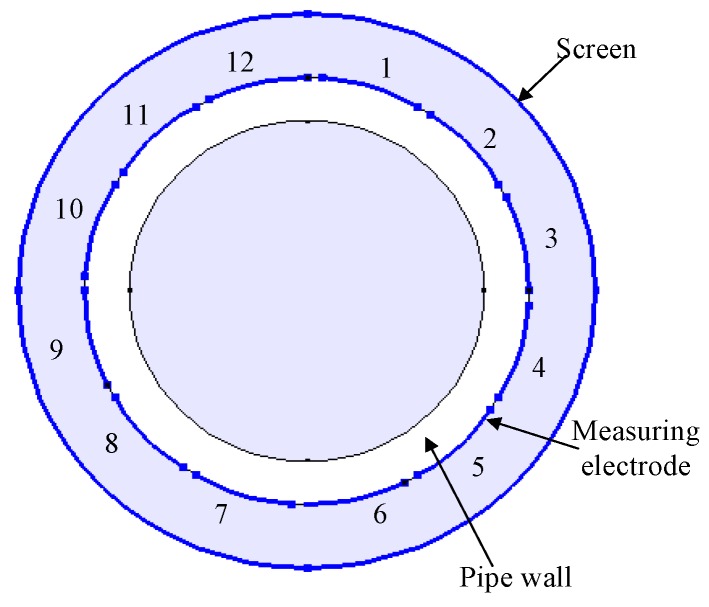
Structure of the capacitance sensor.

In this paper, a typical 12-electrode ECT sensor is used to analyze the effects of the charge distribution on it. The structure of the capacitance sensor is shown in Figure 1. The electrode length is 50 mm, the inner radius of the pipe is 23 mm and the electrode opening angle is 26°. The physical principle of ECT is that different media of multiphase flow have different dielectric constants, thus the equivalent dielectric constant would correspondingly change with the component concentration and distribution of each phase in the pipes, as do the capacitances between the electrodes. There are 66 capacitance values can to be obtained from 12 ECT electrodes: 1–2, 1–3, …, 1–12; 2–3, 2–4…, 2–12;…, 11–12. The media distribution in the pipes can be calculated from the 66 capacitance values. 

ECT consists of two parts: the forward problem and the inverse problem [22]. The forward problem is to determine interelectrode capacitances from the medium spatial distribution [25]. The inverse problem is to estimate dielectric constant field distribution by the measuring capacitance value, namely the image reconstruction.

**Figure 2:**
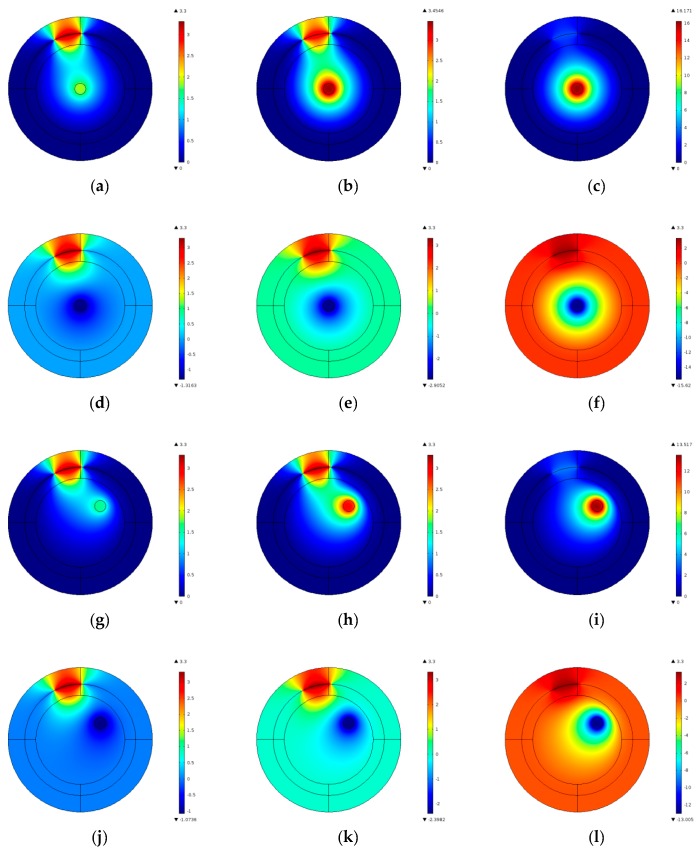
The electric potential under different cases. (**a**) 5 × 10^−9^ C/m^3^; (**b**) 1 × 10^−8^ C/m^3^; (**c**) 5 × 10^−8^ C/m^3^; (**d**) −5 × 10^−9^ C/m^3^; (**e**) −1 × 10^−8^ C/m^3^; (**f**) −5 × 10^−8^ C/m^3^; (**g**) 5 × 10^−9^ C/m^3^; (**h**) 1 × 10^−8^ C/m^3^; (**i**) 5 × 10^−8^ C/m^3^; (**j**) −5 × 10^−9^ C/m^3^; (**k**) −1 × 10^−8^ C/m^3^; (**l**) −5 × 10^−8^ C/m^3^.

**Figure 3:**
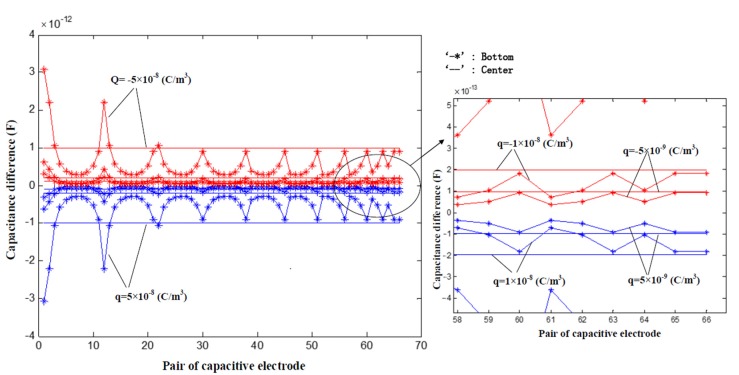
Capacitance differences of the 66 pairs electrodes with or without electrification phenomenon.

The effects of the electrification phenomenon on ECT measurement are investigated by using a finite element method. The entire region inside the screen is divided into 3384 piecewise triangular elements, including 1180 elements within the inner pipe. The low and high relative permittivity for calibration materials inside the sensor are 1.0 and 4.0, respectively. In reference [14], the author drew a conclusion that the greater capacitance differences are produced on the electrode-pairs whose detection electrodes are closer to the charge. Among these electrode-pairs, the greatest electrostatic effect exists on the adjacent ones near the charge. In this paper, the charge density (*q*) of the charged region are set as ±5 × 10^−9^ C/m^3^, ±1 × 10^−8^ C/m^3^ and ±5 × 10^−8^ C/m^3^ in the two-dimensional model, and the charged objects are located in the center of the pipe and in the bottom of the pipe, respectively. The diameter of the charged region is 5 mm. Electric field distributions under different cases (the electrode 12 as the source electrode for example, and the excitation voltage is 3.3 V) are shown in Figure 2. Figure 3 shows the capacitance differences of the 66 electrode pairs with or without the electrification phenomenon. 

From Figure 2 and Figure 3, three main conclusions can be obtained: (1) Capacitance differences are negative under the cases of the positive charged object, and capacitance differences are positively under the cases of the negatively charged object; (2) When the charged object is located in the center of the pipe, the capacitance differences between different electrodes pairs are almost the same. When the charged object is located at the bottom of the pipe, the capacitance differences between different electrodes pairs varies widely, and the greater capacitance differences are produced on the electrode-pairs whose detection electrodes are closer to the charge; (3) The larger the charged carried by the object, the greater the capacitance difference.

In most cases, the normalized linear model of ECT can be expressed as:

(1) C=SG+E

where **C** is the normalized capacitance vector, **G** is the normalized permittivity vector. **E** is the error caused by linear simplification. **S** is a Jacobian matrix, representing the relationship between **C** and **G**, and it is the sensitivity matrix in most image reconstruction methods. The sensitivity matrix is constructed by subdividing the imaging area into small pixels and determining the change in capacitance of each electrode pair due to a small perturbation of the permittivity in each pixel with respect to the empty field. However, through the analysis of the Section 2, the value of **C** is not only affected by the perturbation of the permittivity, but also affected by the perturbation of the charge distribution. In this paper, the neural network algorithm which can construct a mapping relationship between **C** and **G** by using machine learning is investigated with the charging phenomenon, and an improved ELM method is presented to provide a stable nonlinear mapping model between **C** and **G** with good robustness. 

## 3. AST-ELM Image Reconstructed Method

With its fast learning speed, ELM can solve the inverse problem of ECT in a very short period of time. The extreme learning machine (ELM) [24,26] proposed by Guang-Bin Huang is originally developed for the single-hidden layer feed-forward networks (SLFNs) and then extended to the generalized SLENs. 

As for *N* different samples $\left(x_{i},t_{i}\right)$, where $x_{i}={\left[x_{i1},x_{i2},\ldots ,x_{in}\right]}^{T}\in R^{n}$, 1≤i≤N is the network input and $t_{i}={\left[t_{i1},t_{i2},\ldots ,t_{im}\right]}^{T}\in R^{m}$ is the output, the model of SLFNs with $\tilde{N}$ hidden layer nodes and activation function g(x) can be simplified as:

(2) Hβ=T

where $H(a_{1},\cdots a_{\tilde{N}},b_{1},\cdots b_{\tilde{N}},x_{1},\cdots x_{N})={\left[\begin{array}{ccc} g(a_{1}\cdot x_{1}+b_{1}) & \cdots & g(a_{\tilde{N}}\cdot x_{1}+b_{\tilde{N}})\\ \vdots & \ddots & \vdots \\ g(a_{1}\cdot x_{N}+b_{1}) & \cdots & g(a_{\tilde{N}}\cdot x_{N}+b_{\tilde{N}}) \end{array}\right]}_{N\times \tilde{N}}$, $\beta ={\left[\begin{array}{c} \beta_{1}^{T}\\ \vdots \\ \beta_{\tilde{N}}^{T} \end{array}\right]}_{\tilde{N}\times m}$, $Τ={\left[\begin{array}{c} t_{1}^{T}\\ \vdots \\ t_{N}^{T} \end{array}\right]}_{N\times m}$. Where $a_{i}={\left[a_{i1},a_{i2},\ldots ,a_{in}\right]}^{T}$ is the input weight matrix and $b_{i}$ is the input bias of the *i*th hidden layer node, and $\beta_{i}={\left[\beta_{i1},\beta_{i2},\ldots ,\beta_{im}\right]}^{T}$ is the output weight matrix which connects the *i*th hidden layer node, g(x) is the activation function, which could be chosen as sigmoid, sine, hard limit, triangular basis or radial basis function (RBF). 

The essence of ELM is that with sufficient number of hidden layer nodes, SLFN can approximate any continuous function with random input weight $a_{i}$ and bias $b_{i}$.

In order to achieve a good generalization performance, the number of hidden nodes $\tilde{N}$ is set as $\tilde{N}\leq N$, several methods have been proposed to adjust $\tilde{N}$ by Huang [26]. After input weight and bias being assigned randomly, the parameters of hidden layer matrix **H** can be determined and it need not be tuned. So the training of SLFNs is to calculate the least squares solution of Hβ=T.

The number of the training samples *N* is much less than the pixels of reconstructed image *m* in general, so $\tilde{N}\leq m$. It means that the output weight matrix **β** is an ill-posed, sparse, and non-positive definite matrix. In this paper, the adaptive soft-thresholding (AST) algorithm is applied to achieve a stable mapping model by tuning the output matrix **β**, and it can be expressed as follows:

(3) $$\beta_{0}=H^{+}T$$

(4) $$\beta_{k+1}=P_{\omega }\left(\beta_{k}+\alpha_{k}H^{T}(T-H\beta_{k})\right)$$

(5) $$P_{\omega }\left(u\right)=\{\begin{array}{cc} u-\omega & u\geq \omega \\ 0 & \left|u\right|\leq \omega \\ u+\omega & u\leq -\omega \end{array}$$

where *k* is the *k*th iteration. $H^{+}$ is the Moore–Penrose generalized inverse matrix of H. α is the step-length, $\alpha_{k+1}={\left\|H^{T}e_{k}\right\|}_{2}^{2}/{\left\|HH^{T}e_{k}\right\|}_{2}^{2}$, $e_{k}=T-H\beta_{k}$. The determination of the parameter ω is a critical problem, in this paper, an adaptively method is applied to determine the parameter ω. The basic idea is: if we let the threshold result approximately equals to the multistep iteration result, the result may converge to the solution more quickly. It can be expressed as:

(6) $$\beta_{k+l}\approx P_{\omega }(\beta_{k}+\alpha H^{T}(T-H\beta_{k})),\;l\geq 2$$

Based on the iteration scheme:

(7) $$\beta_{k+l}=\beta_{k}+DH^{T}(T-H\beta_{k})$$

where

(8) $$D=\alpha [I+(I-\alpha H^{T}H)+\cdots +{\left(I-\alpha H^{T}H\right)}^{l-1}]$$

where both **D** and **I** are $\tilde{N}\times \tilde{N}$ matrices.

Let $u={\left(\beta_{k}+\alpha H^{T}\left(T-H\beta_{k}\right)\right)}_{i}$,

(9) $${\left(\beta_{k+l}\right)}_{i}=\{\begin{array}{cc} u-\omega & u-\omega \geq 0\\ 0 & \left|u\right|\leq \omega \\ u+\omega & u+\omega \leq 0 \end{array}$$

Substituting Equations (6) and (7) into Equation (9) gives

(10) $$\left\{ \begin{array}{ll} {\left(\left(D-\alpha I\right)H^{T}\left(T-H\beta_{k}\right)\right)}_{i}=-\omega \mathrm{sign}\left({\left(\beta_{k+l}\right)}_{i}\right), & {\left(\beta_{k+l}\right)}_{i}\neq 0\\ \left|{\left(\left(D-\alpha I\right)H^{T}\left(T-H\beta_{k}\right)\right)}_{i}\right|\leq \omega , & {\left(\beta_{k+l}\right)}_{i}=0 \end{array}\right.$$

Then, we can obtain

(11) $$\omega \geq {\left\|\left(D-\alpha I\right)H^{T}\left(T-H\beta_{k}\right)\right\|}_{2}/{\tilde{N}}^{0.5}$$

**Figure 4:**
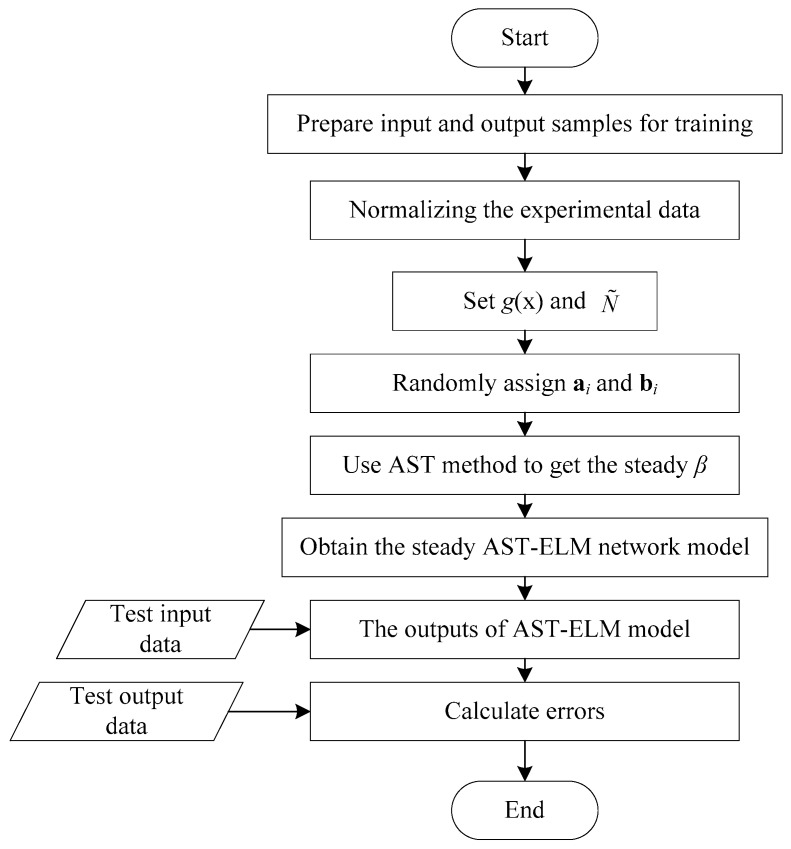
Flowchart of adaptive soft-thresholding extreme learning machine (AST-ELM).

Different ω can be obtained from different l. The optimal l can be determined by experiment, and *l* is set as 6 in this paper [27]. The program flowchart of adaptive soft-thresholding extreme learning machine (AST-ELM) model is shown in Figure 4.

## 4. Simulation and Experimental Results and Discussions

In this section, both simulation and experiment are carried out to evaluate the performance of the AST-ELM method. The quality of the reconstructed images were evaluated in two aspects: relative error and correlation coefficient [28]. Assume that t′ is the medium distribution vector of the reconstructed image, and t is the medium distribution vector of the true image. 

The relative error (δ) of the reconstructed image can be expressed as:

(12) δ=‖t′−t‖/‖t‖×100%

The correlation coefficient (r) can be expressed as:

(13) $$r=\frac{\sum_{j=1}^{m}\left(t\prime_{j}-\bar{t}\prime_{j})(t_{j}-\bar{t}\right)}{\sqrt{\sum_{j=1}^{m}{\left(t\prime_{j}-\bar{t}\prime_{j}\right)}^{2}\sum_{j=1}^{m}{\left(t_{j}-\bar{t}\right)}^{2}}}\times 100\%$$

where *m* is the pixel numbers of the imaging area, $\bar{t}\prime$ and $\bar{t}$ are the average grayscale values in the reconstructed image and the true image separately.

### 4.1. Simulation

Simulated data are generated through the method described in Section 2. MATLAB software is used for image reconstruction and presentation. The inputs of the AST-ELM model are the capacitances and the outputs are the permittivity distributions of the reconstructed image. The number of hidden layer nodes $\tilde{N}$ is set as 160 and the RBF is chosen as activation function. The number of AST iterations is 10.

In the group of simulations, four typical permittivity distributions in multiphase flows are chosen for numerical simulation with a 12-electrode ECT sensor with 3.3 V voltage measurements. The permittivity of the background and the object are set as 1.0 and 4.0, respectively. Two-hundred and forty groups of these cases (120 groups for charged conditions and 120 groups for uncharged conditions) with their simulated data were obtained by using the finite element method. Forty groups (20 groups for each condition) are chosen as the test data randomly, and the rest of the groups are used as training samples for AST-ELM models. 

**Figure 5:**
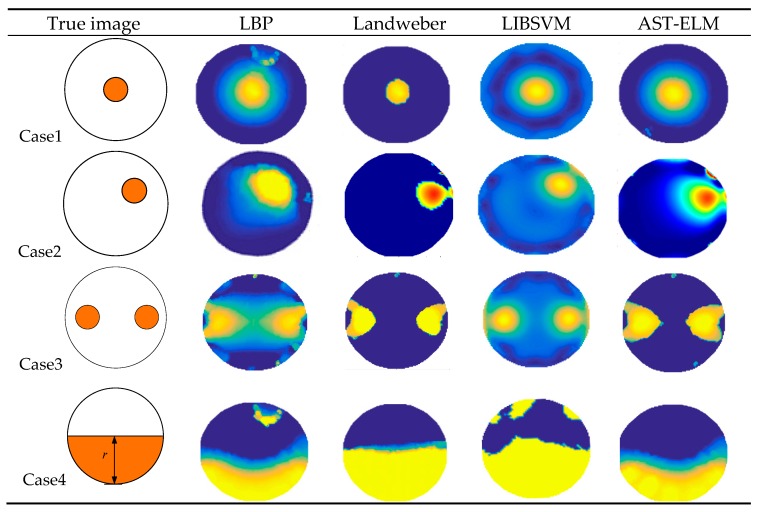
Reconstructed image of uncharged objects.

**Figure 6:**
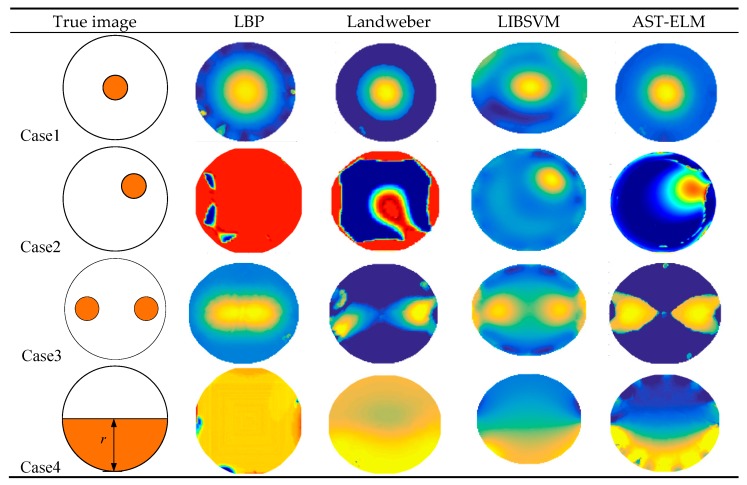
Reconstructed image of charged objects.

As a comparison, the conventional methods of LBP [16], Landweber method (the iteration number and step-length are 200 and 1.2, respectively) and LIBSVM (LIBSVM is a library for Support Vector Machines) methods (the kernel parameter and penalty factor are 2 and 0.5, respectively) are applied to reconstruct these images based on the same simulation data. The reconstructed images of uncharged objects are shown in Figure 5, and the reconstructed images of charged objects (charge density *q* = 1 × 10^−8^ C/m^3^ for example) are shown in Figure 6.

**Figure 7:**
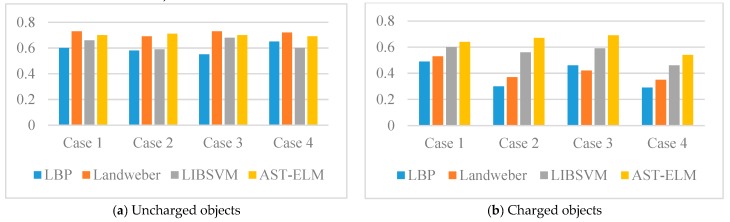
Correlation coefficient (*r*) of simulated data.

**Figure 8:**
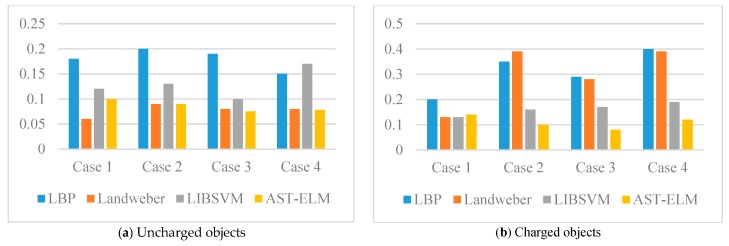
Relative errors (δ) of simulated data.

For quantitatively evaluate the performance of different algorithms, the corresponding correlation coefficient, relative error and reconstruction time of reconstructed images are calculated and displayed in the form of histograms. Please see Figure 7 and Figure 8 and Table 1, (2.50 GHz Intel^®^ Core ^TM^ i5-2450M CPU). 

As shown in Figure 5, the four algorithms are all able to reconstruct the approximate distribution of these four uncharged objects. For the comparison of different methods, the Landweber has the best imaging effects, which is slightly better than that of the AST-ELM method, and the LBP method has the worst imaging effects. The correlation coefficients are calculated and shown in Figure 7a; it shows that the average correlation coefficients of Landweber method is highest (71.5%), which is slightly higher than that of the AST-ELM algorithms (70.2%). The average correlation coefficient of LIBSVM method is 63.2% and LBP has the lowest one (59.5%). The relative errors are calculated and shown in Figure 8a, it shows that the reconstructed images by the Landweber method have smallest average relative errors (0.077) compared with the ones reconstructed by AST-ELM (0.085) , LIBSVM (0.130) and LBP (0.181). They demonstrate that, under the uncharged condition, the Landweber is the most effective algorithm of the three methods. Next to the Landweber method, it is AST-ELM method, and the worst is LBP method.

Figure 6 shows the reconstructed images of four charged objects with the three algorithms, it can be seen that, compared with Figure 5, the imaging qualities of the LBP and Landweber methods have decreased significantly. Most cases are unable to reconstruct the approximate distribution except in case 1, and it can be demonstrated that when the charged object is located in the center of the pipe, the impact on imaging by charged object is small than the other cases. However, for the LIBSVM and AST-ELM method, all cases are able to reconstruct the approximate distribution. The imaging results of AST-ELM method are much better than that of the LBP and Landweber methods. The correlation coefficients are calculated and shown in Figure 7b, it shows that the average correlation coefficients of AST-ELM method is highest (63.5%), which is much higher than that of the rest algorithms, 41.7% for LBP and 38.5% for Landweber. The average correlation coefficient of LIBSVM method is 55.2%. Compared with Figure 7a, the average correlation coefficients of AST-ELM and LIBSVM method are reduced by 6.7% and 8%. However, for the LBP and landweber methods, the amounts of reduction are 29.8% and 21.0%, respectively. The relative errors are calculated and shown in Figure 8b, it shows that the reconstructed images by the AST-ELM method have the smallest average relative errors (0.110) compared with those reconstructed by LIBSVM (0.162), Landweber (0.297) and LBP (0.315). As such, compared with the reconstructed images of the conventional ECT methods, the imaging qualities of the neural network methods are better than that of the other two methods under the charged conditions, especially the proposed AST-ELM method. 

The training time of the two kinds of neural network methods and reconstructed time of the four methods are listed in Table 1. It indicates that the LIBSVM method needs 103.20 s to train the mapping model—which is much longer than that of AST-ELM 3.30 s—so the LIBSVM method will not be investigated in the following part. These simulation results indicate that the AST-ELM method can provide a superior solution to eliminate the influence of electrification phenomenon on ECT measurement.

### 4.2. Experimental Result and Discussion

**Figure 9:**
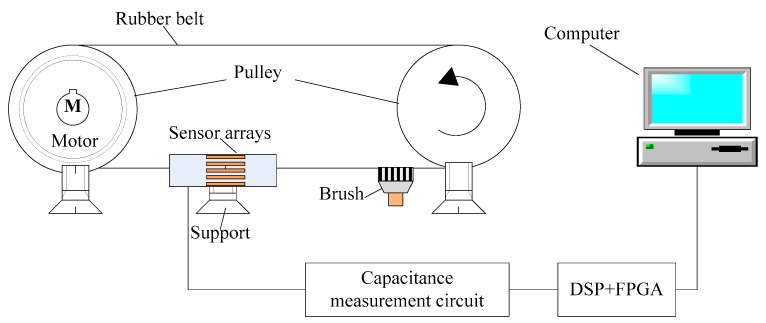
Experimental rig.

A pulley rig with triboelectrification shown schematically in Figure 9 is designed to validate the performance of the proposed method. The test rig is fixed on a steel table which is screwed to the ground by an expansion screw to reduce the vibration of the rig. The rig consists of one electrical motor, two pulleys, one rubber belt, one sensor support, one brush, sensor arrays, signal acquisition and processing unit and a computer. 

When power is on, the rubber belt is moving along with the two pulleys which can be driven by an electric motor, and the speed of the motor can be controlled by a variable-frequency drive (VFD). During the experiments, the rolling rubber belt is used to simulate the moving solid flow. The electrostatic charge can be generated on the belt by rubbing with brush, and the rolling charged belt can be used to simulate the charged object flows. The relative position between the belt and electrodes can be adjusted by the sensor support. 

The design parameters of the sensor arrays are: electrodes number =12, electrode opening angle =26°, electrode thickness =0.2 mm, electrode length =50 mm, pipe inside radius =23 mm, pipe outside radius =25 mm, and the materials of electrodes is copper sheets. The cross-section size of the rubber belt is 14 mm × 10 mm. The investigated field was partitioned into 812 pixels to reconstruct these corresponding objects. The capacitances signals are collected through a C/V (capacitance to voltage) [16], and the sampling frequencies of capacitance signal is 1 MHz. The output signals are transmitted to the signal acquisition and processing unit using shielded cables. In order to meet the high-speed precise data processing of the floating-point and the powerful logic control ability, a hardware platform based on FPGA and DSP (field programmable gate array and digital signal processor) is developed [29]. 

**Figure 10:**
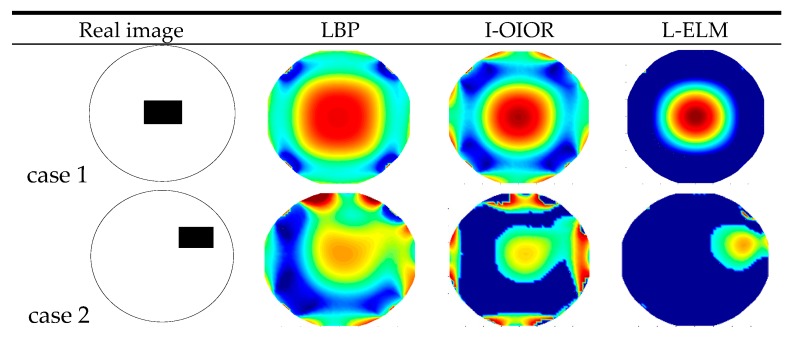

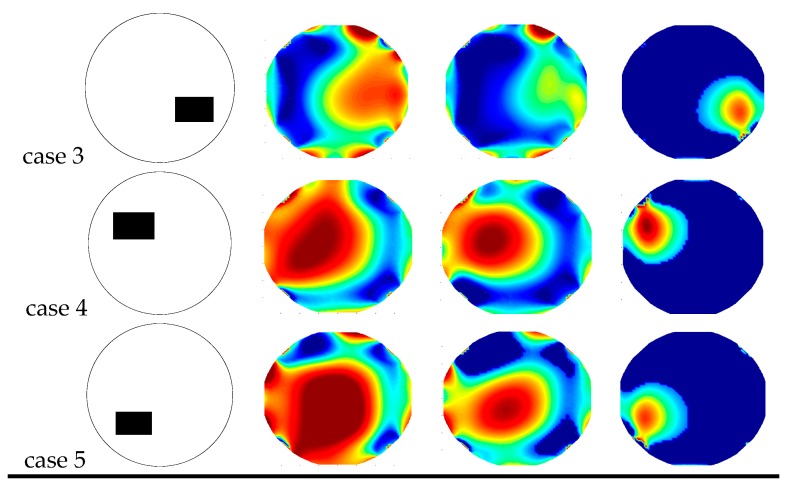
Reconstructed image of experiment data.

**Figure 11:**
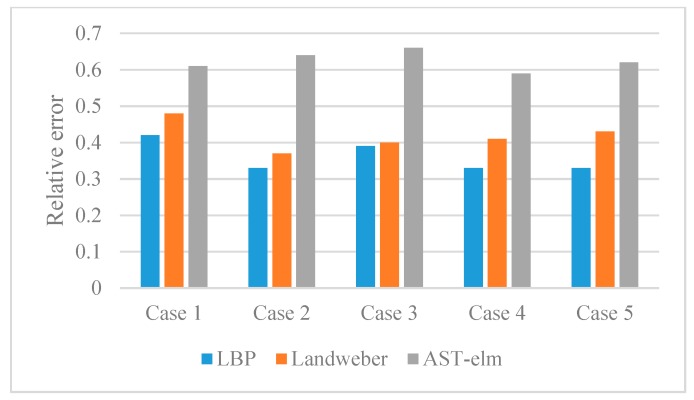
Correlation coefficients (*r*) of experiment data.

**Figure 12:**
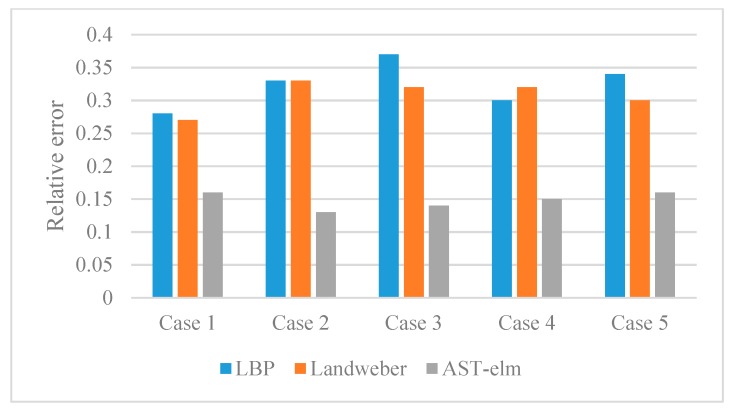
Relative errors (δ) of experiment data.

As described in Figure 9, the rolling rubber belt is used to simulate the moving solid flow, and the rolling charged belt (by rubbing with a brush) is used to simulate the flowing charged object. Five different relative positions with a belt located in the upper right, upper left, lower left, lower right and center of pipe can be obtained by adjusting the sensor support. 170 groups experiment data of these cases are collected for AST-ELM models. Twenty groups are chosen as the test data randomly, and the others are used as training samples. Five representative results among the 20 test groups are analyzed and shown in Figure 10, and the corresponding correlation coefficients and relative errors are shown in Figure 11 and Figure 12. In this part, the LBP and Landweber algorithms are applied to reconstruct these images based on the same measurements as comparisons. 

Figure 10 shows that the AST-ELM method produced the best images, which are closer to the real images. For the other two methods, their reconstructed images are subjected to varying degree of distortion, and the distortions are more likely to appear to the object in a noncentral position; most of the position and size of the belt can’t be recognized in the reconstructed images. Figure 11 shows that the reconstructed images by the AST-ELM method have biggest correlation coefficients (62.4%) compared to those images reconstructed by LBP (41.8%) and Landweber methods (36.0%). Figure 12 shows that the reconstructed images by the AST-ELM method have smallest relative errors (0.148) compared to those images reconstructed by LBP (0.324) and Landweber methods (0.308). So it can be concluded from the experimental results that the imaging qualities of the proposed AST-ELM method can receive a much better performance under the charged conditions, in other words, it can provide a superior solution to eliminate the influence of charged phenomenon on ECT measurement. 

Due to the limited number of independent capacitance measurements and the sampling error of acquisition circuit, it may lead to some limitations on the image reconstruction. For example, the cross-section objects shape of the reconstructed images are oval, however, the true shape of the belt is rectangular. Further research is still needed on image reconstruction.

## 5. Conclusions

In the industrial process detecting system, the charged objects always lead to errors in the practical application of ECT measurements. In this paper, the effects of the charged objects on ECT are investigated, and an improved neural network algorithm of AST-ELM method is presented. A stable and robust nonlinear model mapping the capacitance values to medium spatial distribution by using machine learning rather than electromagnetic-sensitive mechanism. Both simulation and experimental tests are carried out to evaluate this proposed method. Experiments are carried out on a pulley rig with triboelectrification. The results of these simulation and measurement tests illustrated that, under the charging phenomenon, the image reconstruction accuracy by the proposed AST-ELM method has improved greatly in comparison to the conventional ECT method. 

## Figures and Tables

**Table 1:** Reconstruction time.

Methods	LBP	Landweber	LIBSVM		AST-ELM	
Average time (s)	Reconstruction	Reconstruction	Training	Reconstruction	Training	Reconstruction
	0.52	1.09	103.20	0.56	3.30	0.44
